# Transcript profiles of maize embryo sacs and preliminary identification of genes involved in the embryo sac–pollen tube interaction

**DOI:** 10.3389/fpls.2014.00702

**Published:** 2014-12-17

**Authors:** Shuai Shuai Wang, Fang Wang, Su Jian Tan, Ming Xiu Wang, Na Sui, Xian Sheng Zhang

**Affiliations:** ^1^College of Animal Science and Veterinary Medicine, Shandong Agricultural UniversityTai'an, China; ^2^State Key Laboratory of Crop Biology, Shandong Key Laboratory of Crop Biology, College of Life Sciences, Shandong Agricultural UniversityTai'an, China; ^3^College of Life Sciences, Shandong Normal UniversityJi'nan, China

**Keywords:** transcript profiles, embryo sac, pollen tube, interaction, maize

## Abstract

The embryo sac, the female gametophyte of flowering plants, plays important roles in the pollination and fertilization process. Maize (*Zea mays* L.) is a model monocot, but little is known about the interactions between its embryo sac and the pollen tube. In this study, we compared the transcript profiles of mature embryo sacs, mature embryo sacs 14–16 h after pollination, and mature nucelli. Comparing the transcript profiles of the embryo sacs before and after the entry of the pollen tube, we identified 3467 differentially expressed transcripts (3382 differentially expressed genes; DEGs). The DEGs were grouped into 22 functional categories. Among the DEGs, 221 genes were induced upon the entry of the pollen tube, and many of them encoded proteins involved in RNA binding, processing, and transcription, signaling, miscellaneous enzyme family processes, and lipid metabolism processes. Genes in the DEG dataset were grouped into 17 classes in a gene ontology enrichment analysis. The DEGs included many genes encoding proteins involved in protein amino acid phosphorylation and protein ubiquitination, implying that these processes might play important roles in the embryo sac–pollen tube interaction. Additionally, our analyses indicate that the expression of 112 genes encoding cysteine-rich proteins (CRPs) is induced during pollination and fertilization. The CRPs likely regulate pollen tube guidance and embryo sac development. These results provide important information on the genes involved in the embryo sac–pollen tube interaction in maize.

## Introduction

Double fertilization involves a complex mechanism in flowering plants. This process begins when the male gametophyte (pollen) produced by anthers reaches the stigma of the pistil. The pollen grain becomes hydrated on the pistil, and germinates a pollen tube that grows directionally through the stigma and style to enter the ovary, if the pollen tube is recognized by female saprophytic tissue. During this process, the pollen tube–pistil interaction allows the pollen tube to grow along the style and transmission tissue, and signals derived from the embryo sac guide the pollen tube into the micropyle (Johnson and Preuss, 2002; Sanchez et al., 2004; Higashiyama and Hamamura, 2008). The embryo sac consists of only a few cells embedded in the ovule tissue of the pistil. Therefore, it is very difficult to analyze the interaction between the pollen tube and the embryo sac.

The embryo sac usually contains four types of cells: the synergids, the egg cell, the central cell, and the antipodal cells. Genome-wide transcriptome analyses have been used to identify genes with potential functions in the embryo sac. To date, genome-wide transcriptome analyses of embryo sacs have been conducted for *Arabidopsis*, wheat, and maize. The *Arabidopsis* embryo sac dataset was obtained from cDNA subtraction and microarray analyses of embryo sacs at different developmental stages (Yu et al., 2005). An analysis of a wheat cDNA library of embryo sacs revealed 404 egg-expressed genes (Sprunck et al., 2005). In maize, 3850 embryo-sac-specific transcripts and 963 egg-cell-specific transcripts were detected in embryo sac and egg cell cDNA libraries (Yang et al., 2006). Whole-genome tiling microarray and high-throughput cDNA sequencing analyses identified genes expressed specifically in the ovules of wild-type in *Arabidopsis*, such as *myb98* and *dif1* (Jones-Rhoades et al., 2007). Recently, the gene transcript profiles of synergid cells, egg cells, and central cells in the mature *Arabidopsis* female gametophyte were analyzed via laser-assisted microdissection of individual cells and Affymetrix ATH1 GeneChip expression analyses (Wuest et al., 2010).

The genes identified in embryo sac have been shown to have functions in pollen tube guidance. For example, the synergids have been shown to play a direct role in pollen tube attraction and guidance in the ovules of *Arabidopsis, Torenia fournieri*, and maize (Higashiyama, 2002; Punwani et al., 2008). The FERONIA (FER) protein is expressed in the synergids, and localizes at the filiform apparatus in *Arabidopsis*. In *fer* mutants, wild-type pollen tubes fail to arrest growth and invade the female gametophyte without releasing sperms (Huck et al., 2003; Rotman et al., 2003). MYB98 is a transcription factor that is expressed preferentially in the synergid cells of *Arabidopsis*. The *myb98* mutant shows defective organization of the filiform apparatus in synergid cells and defective micropylar guidance of pollen tubes, indicating that the proper function of synergid cells is essential for micropylar guidance (Kasahara et al., 2005). The maize gene *ZmEA1* is specifically expressed in the egg apparatus. The ZmEA1 protein was shown to attract maize pollen tubes directly *in vitro*. The pollen tube could not penetrate the intercellular space of the micropyle in *ZmEA1*-knockdown plants, suggesting that ZmEA1 might be involved in guiding the pollen tube to the micropyle (Dresselhaus and Márton, 2009; Márton and Dresselhaus, 2010). In *T. fournieri, LURE1* and *LURE2* encode micropylar pollen-tube attractants derived from the synergids (Okuda et al., 2009). As well as the synergids, other female gametophyte cells also function in pollen tube guidance. AtCCG is a putative transcriptional regulator expressed in the central cell. The *ccg* mutant abolishes micropylar guidance, demonstrating that the central cell is required to guide the pollen tube to the micropyle (Chen et al., 2007). GEX3, a protein localized in the plasma membrane of the male gametophyte and in the egg cell of the female gametophyte, is also essential for pollen tube guidance (Alandete-Saez et al., 2008).

Analyses of the maize genome can provide important information on the molecular mechanism of the embryo sac–pollen tube interaction (Dresselhaus et al., 2011). The quantitative RNA-seq analysis of embryo sacs was preformed and used to identify genome features with differential expression between the gametophytes and sporophytic tissues, including protein-coding gene families, duplicated genes and previously unannotated genes (Chettoor et al., 2014). However, the genes involved in post-pollination have been never studied at a comprehensive level, and they may be involved in the embryo sac–pollen tube interaction in maize. In this study, the transcriptomes of mature embryo sacs, embryo sacs 14–16 h after pollination, and mature nucelli of maize were analyzed by RNA-sequencing (RNA-seq). By comparing the transcriptomes of the maize embryo sac before and after entry of the pollen tube, we identified 3467 differentially expressed transcripts (3382 differentially expressed genes; DEGs), including well-known and new genes involved in pollen tube guidance. The results of this study provide new insights into the complex regulatory networks underlying the embryo sac–pollen tube interaction in maize.

## Materials and methods

### Isolation of female gametophytes and nucelli

The maize inbred line Q319 was used for RNA-seq analyses. The maize plants were cultivated in a field at the Experimental Station of Shandong Agricultural University. Before the experiment, whole ears were covered with paper bags to avoid cross-pollination after pollen release. We sampled the ovaries before and after pollination. Mature silks were lightly pollinated with approximately 0.3 g fresh mature pollen grains at 10:00 AM. Then, 14 h later, female gametophytes were isolated by enzymatic immersion of ovule slices, as described by Yang et al. (2006). The nucelli were cut directly from the ovule. The female gametophytes and nucelli were frozen in liquid nitrogen and stored at −80°C until RNA extraction.

### RNA extraction and library construction

Total RNAs were extracted from embryo sacs and nucelli using an RNeasy Plus Micro kit (Qiagen, Valencia, CA, USA). At least 500 ng total RNA was extracted from each material used for library construction. First, mRNAs extracted from each material were enriched by using oligo(dT) magnetic beads (Illumina, San Diego, CA, USA). The purity and quantity of total RNA was checked (Additional file 1). Then, mRNAs were further enriched by removing rRNAs from the total RNA. The mRNAs were broken into short fragments (approximately 200 bp) in fragmentation buffer. First-strand cDNA was synthesized with random hexamer primers using the short fragments as templates. Then, dNTPs, buffer, DNA polymerase I, and RNase H were added to synthesize the second strand. The double-stranded cDNAs were purified with a QiaQuick PCR Extraction kit and washed with EB buffer for end repair and single nucleotide A (adenine) addition. Finally, sequencing adaptors were connected to the fragments. The fragments were purified by agarose gel electrophoresis and enriched by PCR amplification.

### Illumina sequencing and data analysis

Each library was sequenced using the Illumina HiSeq™ 2000 system at the Beijing Genomics Institute (Shenzhen, China). Single-end 49-bp fragment reads were collected. The sequencing quality of raw data was analyzed by the Illumina Genome Analysis Pipeline, version 1.6. Reads with more than 10% unknown bases, low-quality raw reads, and reads with adaptors were excluded. The remaining clean reads were used for subsequent analyses. All sequence data have been submitted to the RNA-seq database under accession number GSE57075. The clean reads were aligned to the AGPv2 maize B73 reference genome using the short oligo-nucleotide alignment program v.2 (SOAP2) (Li et al., 2009). Mismatches of less than two bases were allowed in this process. According to the alignment, clean reads were divided into unmapped reads, multi-position matched reads, and unique matched reads. For all mapped transcripts with unique matched reads, the original digital gene expression levels were calculated using reads per kilobase per million (RPKM) method (Mortazavi et al., 2008).

We used edgR analysis software (Robinson et al., 2010) to identify preferentially expressed genes in the ES (EPGs) and DEGs before and after pollination. The DEGs (ES *vs*. Nu, and ESP *vs*. ES) were identified based on a fold change of ≥2 and a false discovery rate (FDR) of <1E-05. Gene annotations were obtained from the maize B73 sequence AGP V2 5b.60 (http://www.maizesequence.org/index.html).

Genes were classified using MapMan software. The gene ontology (GO) analysis was conducted using the singular enrichment analysis tool (http://bioinfo.cau.edu.cn/agriGO/analysis.php). The sub-cellular location of each gene was predicted using TargetP 1.1. (http://www.cbs.dtu.dk/services/TargetP/). The signal peptide analysis was performed using TargetP 1.1 (http://www.cbs.dtu.dk/services/SignalP//). Protein domains were predicted using the pfam tool (http://pfam.sanger.ac.uk/search?tab=searchSequenceBlock), and the cysteine-rich proteins (CRPs) were predicted and analyzed as described in Xu et al. (2012).

### Laser scanning confocal microscopy (LSCM) observations of maize embryo sac

At 14 h after pollination, mature ovaries were collected from the middle of the ears, immersed in ice-cold FAA fixative, and then placed under vacuum for 20–30 min. Then, the ovaries were immersed in fresh fixative and again placed under vacuum for 20–30 min. The samples were incubated at 4°C for 24–48 h, and then whole nucelli were isolated by manual microdissection. The nucelli were rehydrated in a graded ethanol series (v/v: 50, 30, and 10%) for 30 min at each step, and then washed with 100% distilled water three times for at least 30 min per wash. Then, the samples were stained with 4% (w/v) sucrose red for 12–48 h. The dye was removed from the samples by three washes in 100% distilled water. The materials were again dehydrated in a graded ethanol series (v/v: 10, 30, 50, 70, 90, and 100%) for 30 min at each step, and then finally subjected to three 30-min washes with 100% ethanol. The samples were treated with 50% methyl salicylate (methyl salicylate: ethanol = 1:1) for 2 h, and then with 100% methyl salicylate for 12–48 h before observation under a Zeiss LSM-510 confocal microscope (excitation wavelength: 488 nm; filters: BP505-550; Carl Zeiss, Jena, Germany).

### *In situ* hybridization

*In situ* hybridization was performed as described by Guo et al. (2010). Briefly, the maize ovules were fixed in FAA (v/v: 3.7% formaldehyde, 5% acetic acid, 50% alcohol) overnight at 4°C. Then, the samples were dehydrated and embedded in paraplast resin (Sigma, St. Louis, MO, USA) and cut into 8-μm-thick sections. Antisense and sense RNA probes were labeled *in vitro* from cDNA fragments (448 bp fragments at the 5′-UTR of *GRMZM2G165083*, 510 bp fragments at the 5′-UTR of *GRMZM2G165084*) using a digoxigenin RNA labeling kit (Boehringer Mannheim, Mannheim, Germany). They were then hydrolyzed to 200 nucleotides of average length by alkali treatment. After pretreatment, slides were hybridized with the probe (200 ng probe/mL hybridization solution containing 50% v/v formamide) at 47°C overnight. For the detection of hybridized signals, hybridized probes were used with ananti-digoxigenin antibody conjugated with alkaline phosphatase (DIG Nucleic Acid Detection Kit, BoehringerMannheim). Photographs were taken using the Olympus BH-2 microscope.

### RT-qPCR analyses

Total RNA was extracted using the method described above and then treated with RNase-free DNase I (Promega, Madison, WI, USA) to eliminate genomic DNA. Total RNA (4 μg) was used to synthesize cDNA with oligo (dT) primers using M-MLV reverse transcriptase (Promega), according to the manufacturer's instructions. The qRT-PCR analyses were carried out using SYBR Green Real-time PCR Master Mix (Toyobo, Osaka, Japan) with a Bio-Rad CFX96 Real-Time Detection System. For each gene analyzed by qRT-PCR, three biological replicates were analyzed. In each qRT-PCR run, 18S rRNA was used to normalize mRNA levels. Quantitative variations among different replicates were calculated using the delta-delta threshold cycle relative quantification method. The primers used for qRT-PCR are listed in Additional file 2.

### Semi-quantitative RT-PCR analyses

Total RNAs were isolated from roots, stems, leaves, pollen grains, and ovaries with TRIzol reagent (Invitrogen, Carlsbad, CA, USA), according to the manufacturer's instructions. The RNAs were treated with DNase I to eliminate genomic DNA (RNase-free; Promega). Details of the primers and cycle conditions are available on request. These experiments were independently replicated at least three times under identical conditions. The transcript level of 18s rRNA (detected using the primers 5′-CGGCTACCACATCCAAGGAA-3′ and 5′-TGTCACTACCTCCCCGTGTCA-3′) was analyzed as the internal control, and was used to normalize all data.

## Results and discussion

### Transcriptome analysis of maize embryo sac after pollination

**Figure 1:**
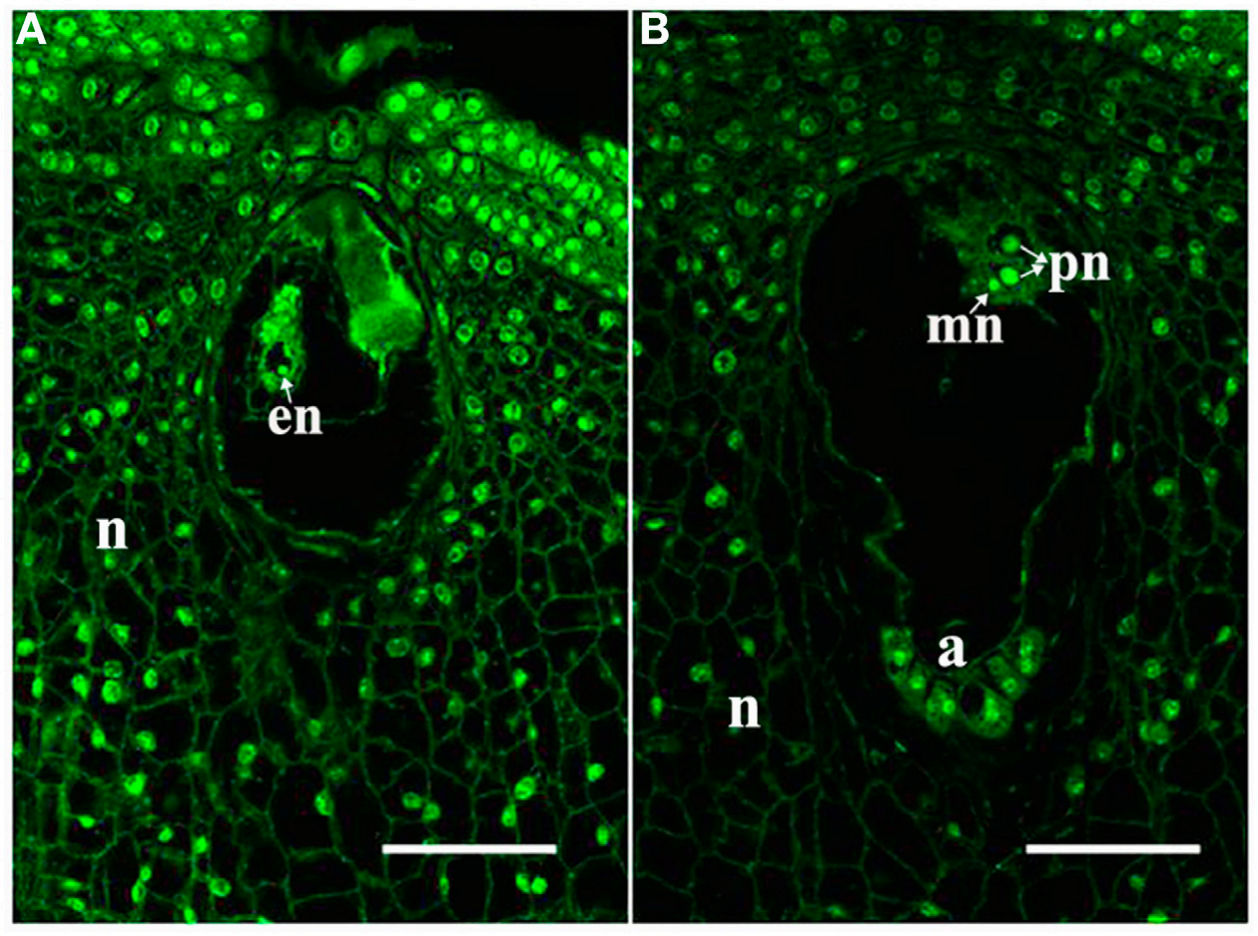
**Samples used for RNA-seq analyses**. **(A)** ES with an egg; **(B)** ESP with a central cell, male nucleus (mn) moving to female nucleus (arrowheads). a, antipodal cells; en, egg nucleus; pn, polar nucleus; n, nucellus. Scale bars = 50 μm.

In the maize cultivar Q319, the time from pollination until the arrival of the pollen tube at the embryo sac is approximately 14 h. Therefore, we identified the ES (embryo sac at anthesis) and the ESP (embryo sac at 14 h after pollination) by laser scanning confocal microscope observation. In the ES, the egg nucleus was present (Figure 1A). In the ESP, the male nucleus was close to the polar nucleus. It suggested that the female and male gametes interact in the ESP (Figure 1B).

To identify the genes involved in the embryo sac–pollen tube interaction, the total RNAs were isolated from the ES and ESP and subjected to RNA-seq analyses. The total RNAs of the nucellus (Nu) at the ES stage was also sequenced as a control. The mRNAs of each of the three tissues were obtained from independent biological samples, and were used to construct cDNA libraries, which were then sequenced by the Illumina HiSeq 2000 system. After quality control of sequences (Additional file 3) and removal of ‘dirty’ raw reads (see Materials and Methods), the number of purity-filtered reads varied from 6,356,811 to 9,049,923 per library (Additional file 3). The tag density was sufficient for quantitative analyses of gene expression. The Spearman's and Pearson's r values and the slope (k) values were close to 1, indicating good reproducibility among tissues (Additional file 4).

**Figure 2:**
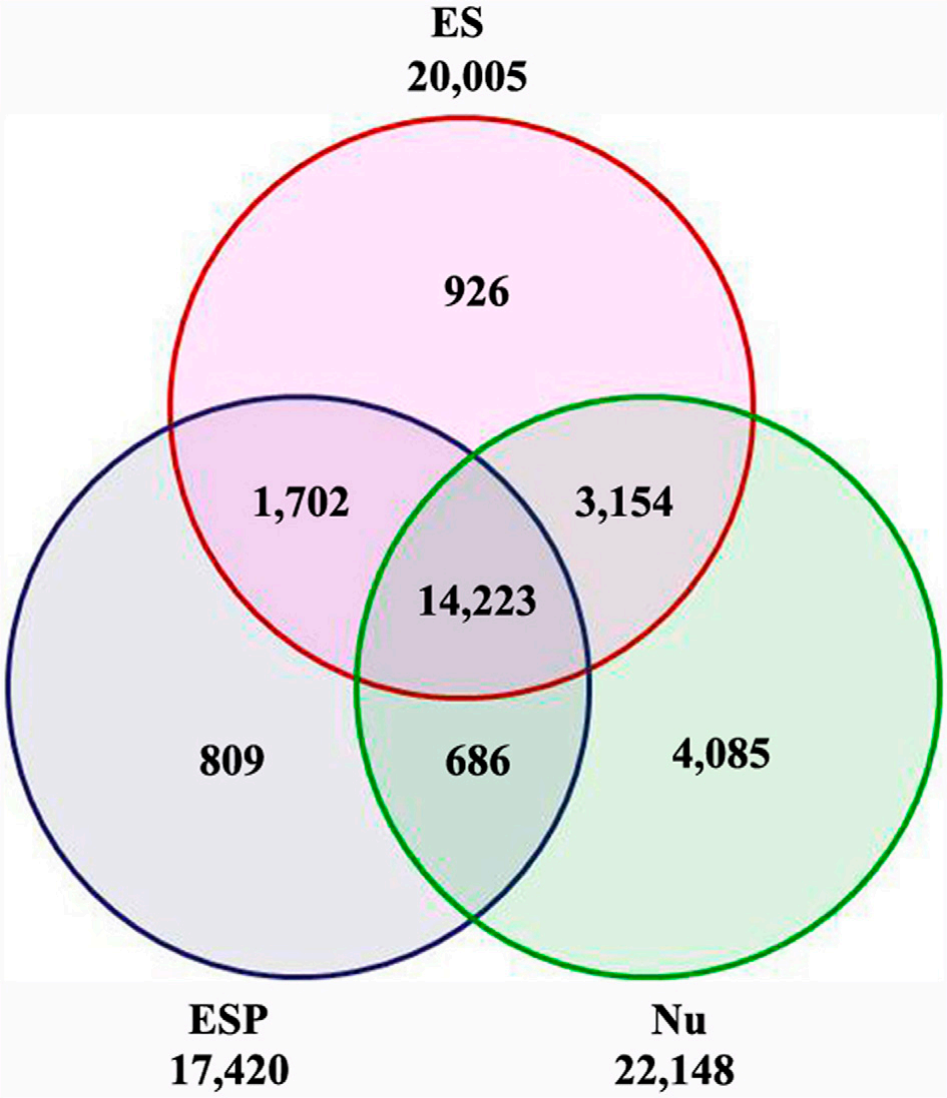
**Venn diagram showing number of transcripts detected in ES, ESP, and Nu**.

To identify the genes corresponding to the reads in each library, the filtered clean reads were mapped to version 2 of the maize B73 reference genome (AGPv2, http://www.maizesequence.org) using SOAP2 software. To make the libraries meaningful, transcripts with RPKM values of less than 1 were eliminated before further statistical analyses. Finally, 20,005 transcripts (18,257 genes) in the ES, 22,148 transcripts (19,354 genes) in the Nu, and 17,420 transcripts (16,503 genes) in the ESP were detected (Figure 2, Additional file 5). Among the 20,005 transcripts detected in the ES, approximately 80% (15,925/20,005) were also detected in the ESP. Of them, 809 transcripts (803 genes) were specifically expressed in the ESP (Figure 2, Additional file 6). In previous studies, many genes have been identified in cell-specific groups of embryo sac (Lê et al., 2005; Yu et al., 2005; Yang et al., 2006; Jones-Rhoades et al., 2007; Wuest et al., 2010; Chettoor et al., 2014). Compared with those data, we identified homologs of 176 genes in other species, which were specifically expressed in egg, central cell or synergids (Additional file 6). The information in this study might provide the important gene resources for the network underlying the embryo sac-pollen tube interaction.

### Changes in transcript profile of the maize embryo sac after entry of the pollen tube

In total, 3607 transcripts (3467 genes) were identified as EPGs. These transcripts above 1 RPKM were identified as being differentially expressed in the ES (*vs*. Nu) based on a -fold change of ≥2 and a FDR of < 1E-05 (Additional file 7). The quantitative RNA-seq analysis of maize embryo sacs showed that the expression levels of 5011 genes above 0.1 FPKM were 2 fold higher than in ovules with the embryo sacs removed, mature pollen, and seedlings (Chettoor et al., 2014). The 806 genes in EPGs also appeared in the data of Chettoor et al. (2014), suggesting that our data was credible (Additional file 7).

To identify DEGs after entry of the pollen tube, we analyzed digital gene expression using edgR software. The DEGs were identified based on a -fold change of ≥2 and a FDR of < 1E-05. Transcripts with RPKM values of less than 1 were eliminated. In total, 1627 transcripts (1619 genes) were identified as significantly up-regulated and 1840 transcripts (1773 genes) were identified as significantly down-regulated in the ESP, compared with the ES (Additional file 8).

**Figure 3:**
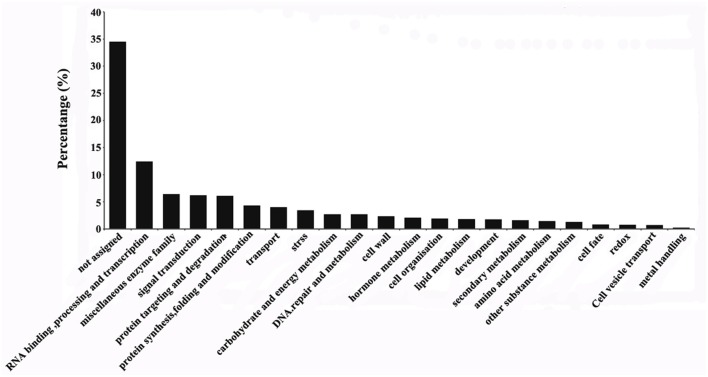
**Classification of differentially expressed genes in the ES before and after entry of the pollen tube**. Classification results were obtained using MapMan software.

The 3467 DEGs were grouped into 21 functional categories and one unassigned category using MapMan software (Figure 3). The four functional categories with the highest proportions of DEGs were RNA binding, processing and transcription (12.5%), miscellaneous enzyme family (6.4%), signaling transduction (6.2%), and protein targeting and degradation (6.1%). Among the DEGs, 34.5% were grouped in the “not assigned” category.

**Figure 4:**
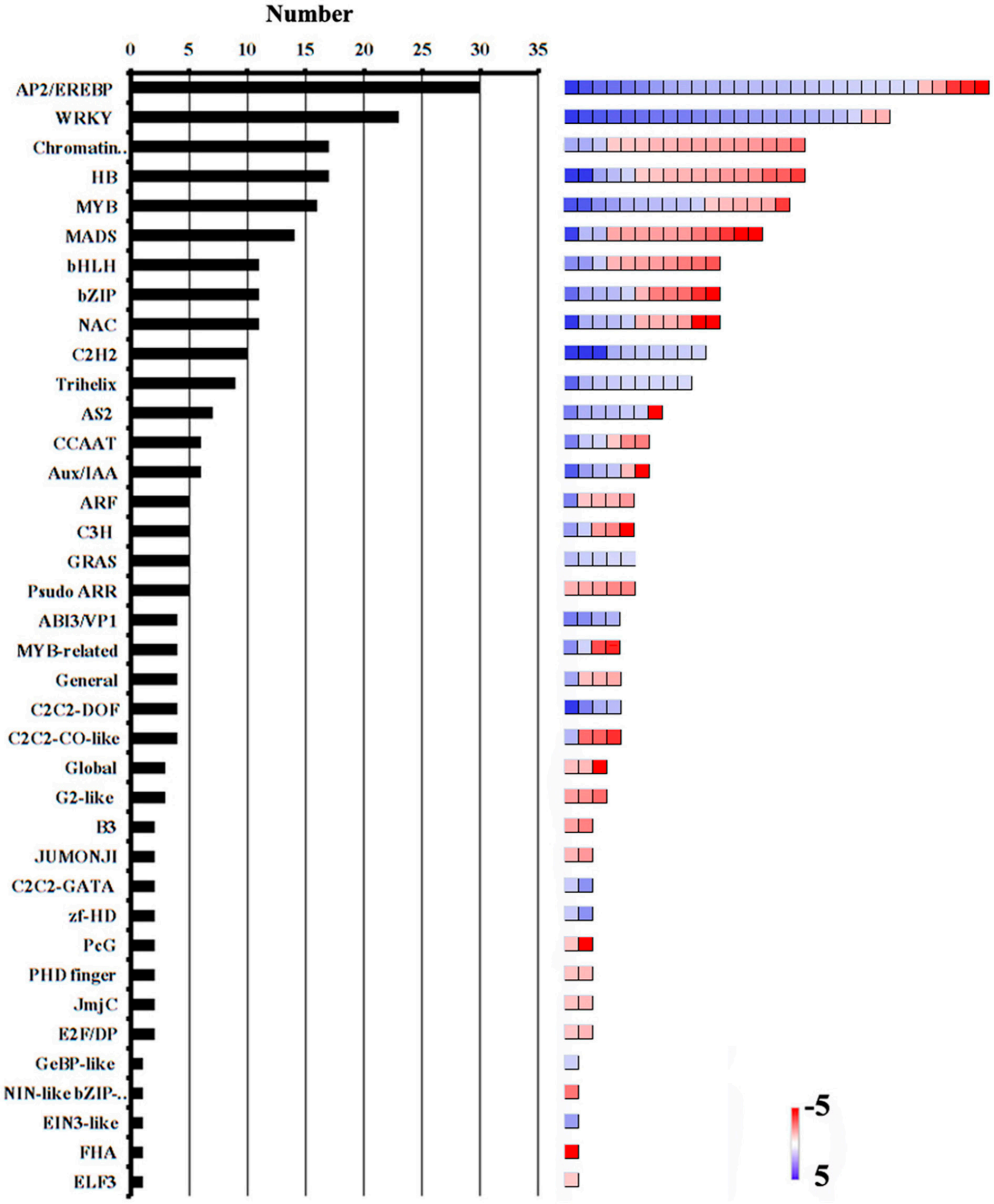
**Abundance of TF families among DEGs**. Number of genes in each category is shown on the left. Color scales (right) show relative transcript abundance of each gene (log_2_-transformed RPKM values) in the given categories.

Transcription factors (TFs) are a class of proteins that regulate gene transcription and expression by recognizing and binding to *cis*-acting elements in the promoters of the target genes. Among the 432 genes involved in RNA binding and processing, and transcription, 380 encoded putative TFs. The most abundant TF families were the APETALA2/ethylene-responsive element binding protein (AP2/EREBP), WRKY, chromatin remodeling, homeobox protein (HB), v-myb avian myeloblastosis viral oncogene homolog (MYB), and mcm1-agamous-deficiens serum response factor (MADS) families (Figure 4, Additional file 9). Many members of these subfamilies are known to play important roles in reproductive processes. For example, the AP2/ERF TFs were shown to regulate plant hormone responses (Nakano et al., 2006; Licausi et al., 2010; Sharoni et al., 2011). Both auxin and ethylene regulate ovary and ovule development, and coordinate the development of male and female gametophytes (Zhang and O'Neill, 1993). *MYB98*, which is specifically expressed in the synergid cells, was shown to have an essential role in pollen tube guidance and in the formation of the filiform apparatus (Kasahara et al., 2005). The MADS-box transcription factor AGL23 was shown to control female gametophyte and embryo development in *Arabidopsis* (Colombo et al., 2008). In addition, AtCCG, which is expressed in the central cell of the ovule, might act as TF to guide the pollen tube to the micropyle in *Arabidopsis* (Chen et al., 2007). In our data, the transcript level of the *AtCCG*-homolog *GRMZM2G307720* was significantly lower in the ESP than in the ES. Considering pollination-regulated the expression of *TF* genes, we suggest that these genes might function in the embryo sac–pollen tube interaction in maize.

In flowering plants, signaling between the pollen tube and the embryo sac is required for fertilization. FER, a putative RLK1-like kinase in *Catharanthus roseus* (CrRLK1L), is localized at the filiform apparatus, and regulates the male–female interaction during pollen tube reception (Escobar-Restrepo et al., 2007). ANXUR1 and ANXUR2 (ANX1, ANX2), which encode the closest homologs of FER-RLK in *Arabidopsis*, were shown to be preferentially expressed in pollen. The pollen tube of *anx1*/*anx2* mutants ruptured before arriving at the egg apparatus, suggesting that ANX1 and ANX2 might function as male factors controlling pollen tube behavior by directing rupture at the appropriate time (Boisson-Dernier et al., 2009). In our data, there were four transcripts (*GRMZM2G100288, GRMZM5G897958, GRMZM2G335052*, and *GRMZM2G16542*) encoding CrRLK1L homologs. Three transcripts (*GRMZM2G100288, GRMZM2G335052* and *GRMZM2G16542*) were up-regulated and one transcript (*GRMZM5G897958*) was down-regulated after pollination. One of them, *GRMZM2G100288*, encodes a protein with moderate similarity (approximately 63%) to FER, suggesting that CrRLK1L homologs might play an important role in the embryo sac–pollen tube interaction in maize.

**Figure 5:**
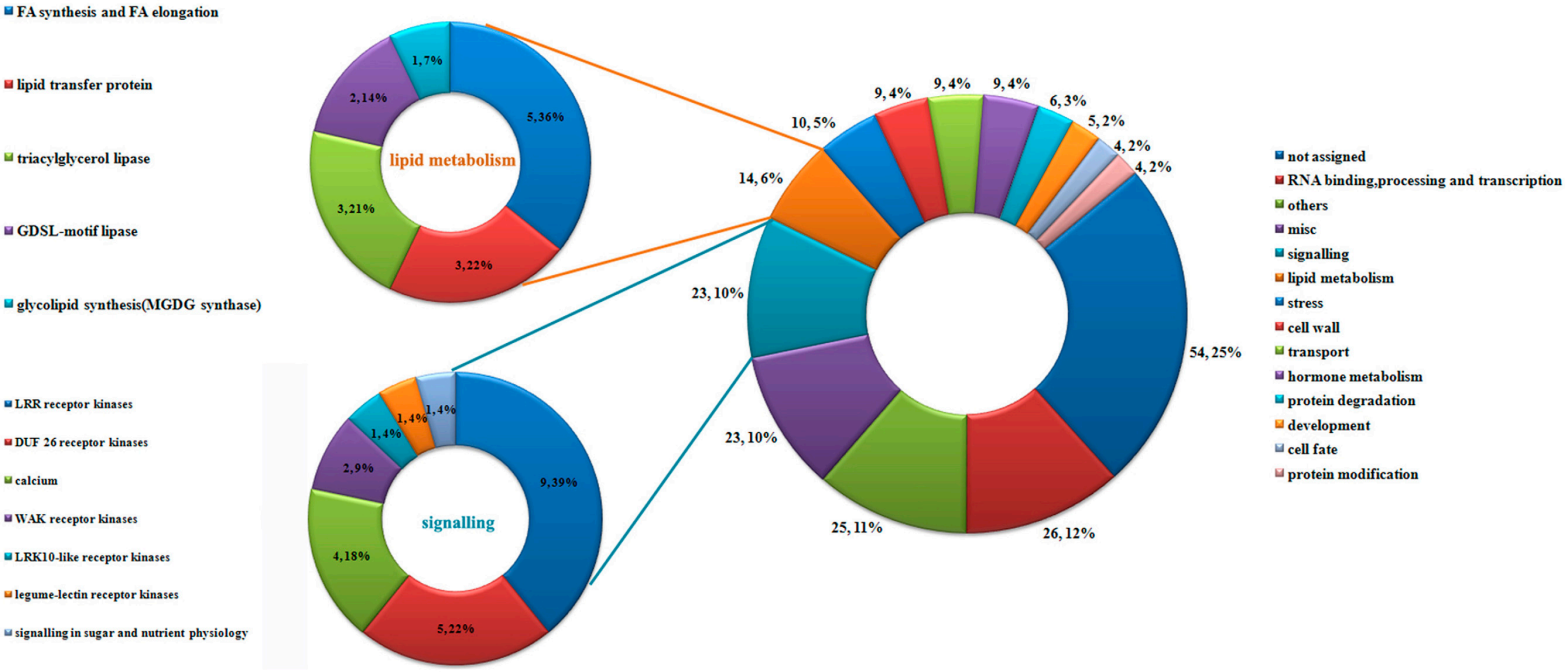
**Classification of genes induced by pollination**. Using MapMan, 221 genes induced by pollination were classified into 14 categories; details of genes involved in lipid metabolism and signaling are shown on the left.

Interestingly, 221 transcripts were expressed exclusively in the ESP (Additional file 10). We defined these 221 transcripts as those that were induced by the entry of the pollen tube. These genes may encode proteins that function in the pollen–embryo sac interaction in maize. Using MapMan software, the 221 transcripts were grouped into 11 functional categories (Figure 5). As shown in Figure 5, the largest category was un-annotated genes (25%), followed by genes involved in RNA binding, processing and transcription (12%), signaling (10%), miscellaneous enzyme family processes (10%) and lipid metabolism (6%).

### Differentially expressed genes encoding proteins with possible roles in the embryo sac–pollen tube interaction

To gain an overall picture of the DEGs, we performed a GO enrichment analysis. The DEGs were grouped into cellular component, molecular function, or biological process categories. The results showed that 17 GO terms were overrepresented among the DEGs, based on a *p* value of < 0.001 and an FDR of ≤0.05 (Table 1). Within the biological process category, there were many DEGs in the response to stress, protein amino acid phosphorylation, and protein ubiquitination subcategories. This result indicated that these pathways and processes might be important in the embryo sac–pollen tube interaction.

**Table 1:** **Over-represented functional GO terms among DEGs in the maize ES before and after entry of the pollen tube**.

**GO Term^a^**	**Term type^a^**	**Query item^b^**	**Query total^c^**	**Bg item^d^**	**Bg total^e^**	***p*-Value^f^**	**FDR^g^**
DNA-dependent DNA replication	P	9	2068	32	39203	3.00E-05	0.012
Negative regulation of molecular function	P	13	2068	66	39203	3.70E-05	0.012
Protein amino acid phosphorylation	P	172	2068	2387	39203	6.00E-05	0.013
L-phenylalanine metabolic process	P	9	2068	34	39203	5.00E-05	0.013
Response to stress	P	214	2068	3059	39203	5.40E-05	0.013
Protein ubiquitination	P	19	2068	139	39203	0.00014	0.026
Transferase activity, transferring glycosyl groups	F	71	2068	747	39203	2.40E-06	0.003
Hydrolase activity, acting on glycosyl bonds	F	74	2068	821	39203	9.60E-06	0.003
Ammonia-lyase activity	F	8	2068	24	39203	2.10E-05	0.0049
Protein serine/threonine kinase activity	F	165	2068	2248	39203	3.30E-05	0.006
ATP binding	F	341	2068	5133	39203	4.30E-05	0.0061
Transcription factor activity	F	108	2068	1390	39203	7.70E-05	0.0083
Calcium ion binding	F	49	2068	534	39203	0.00017	0.016
Ubiquitin-protein ligase activity	F	19	2068	141	39203	0.00017	0.016
Transferase activity, transferring acyl groups other than amino-acyl groups	F	36	2068	357	39203	0.0002	0.017
Identical protein binding	F	13	2068	79	39203	0.00025	0.019
Copper ion binding	F	22	2068	194	39203	0.00067	0.046

a *GO term classifications: P, biological process; C, cellular component; F, molecular function*.

b *Query item number in ES preferentially expressed genes*.

c *Total annotated query item number in agriGO*.

d *Query item number in maize genome version 5a*.

e *Total annotated item number in maize genome version 5a*.

f *Determined by Fisher's exact test*.

g *Determined by the Benjamini–Hochberg–Yekutieli procedure*.

*GO terms with p-value <0.001 and FDR ≤0.05 were regarded as over-represented terms*.

### Protein ubiquitination

The ubiquitin–proteasome system (UPS) consists of five components: ubiquitin, ubiquitin activating enzyme E1, ubiquitin-conjugating enzyme E2, ubiquitin ligase enzyme E3, and proteasomes. The UPS system appears to play a central role in spermatogenesis and fertilization (Nakamura, 2013). Approximately 70 E3 ubiquitin ligases are expressed during spermatogenesis in mice (Hou et al., 2012). In mammals, abnormal spermatozoa are marked with ubiquitin during epididymal passage. Subsequently, most of the ubiquitinated spermatozoa are phagocytosed by the epididymal epithelial cells, indicating that ubiquitin mediates sperm quality control and regulates male fertility (Sutovsky et al., 2001). In flowering plants, there are relatively high levels of protein ubiquitination in generative cells and sperm cells, suggesting that the UPS may be involved in the development of the male gametophyte (Singhmb et al., 2002). The small ubiquitin-like modifier (SUMO) E3 ligase, SAP, and Miz-finger domain-containing protein 1 (SIZ1) maintain the stability and normal function of the mature female gametophyte, which is necessary for pollen tube guidance in *Arabidopsis* (Ling et al., 2012). Previously, we showed that UPS proteins, especially E3 ubiquitin ligases, are involved in the pollen tube–silk interaction in maize (Xu et al., 2013).

The GO analysis revealed that 19 genes related to protein ubiquitination were among the DEGs (Table 1). A further MapMan analysis showed that 17 of these 19 genes encode Ring-type E3 ubiquitin ligases (Additional file 11). These findings suggest that UPS proteins, such as E3 ubiquitin ligases, are likely to be involved in the development of the embryo sac and in the embryo sac–pollen tube interaction in maize.

### Protein amino acid phosphorylation

Protein amino acid phosphorylation is involved in almost all cellular processes. GO enrichment analysis of the DEGs showed that there were 172 genes related to protein amino acid phosphorylation (Table 1, Additional file12), suggesting that many proteins in the embryo sac were phosphorylated after the pollen tube had entered the embryo sac. Interestingly, we found that genes related to protein amino acid phosphorylation were enriched in Nu, but not in ES. This finding suggests that there was a lower level of protein phosphorylation in the mature embryo sac than in the sporophyte. Thus, it is likely that protein phosphorylation events are involved in the embryo sac–pollen tube interaction.

**Figure 6:**
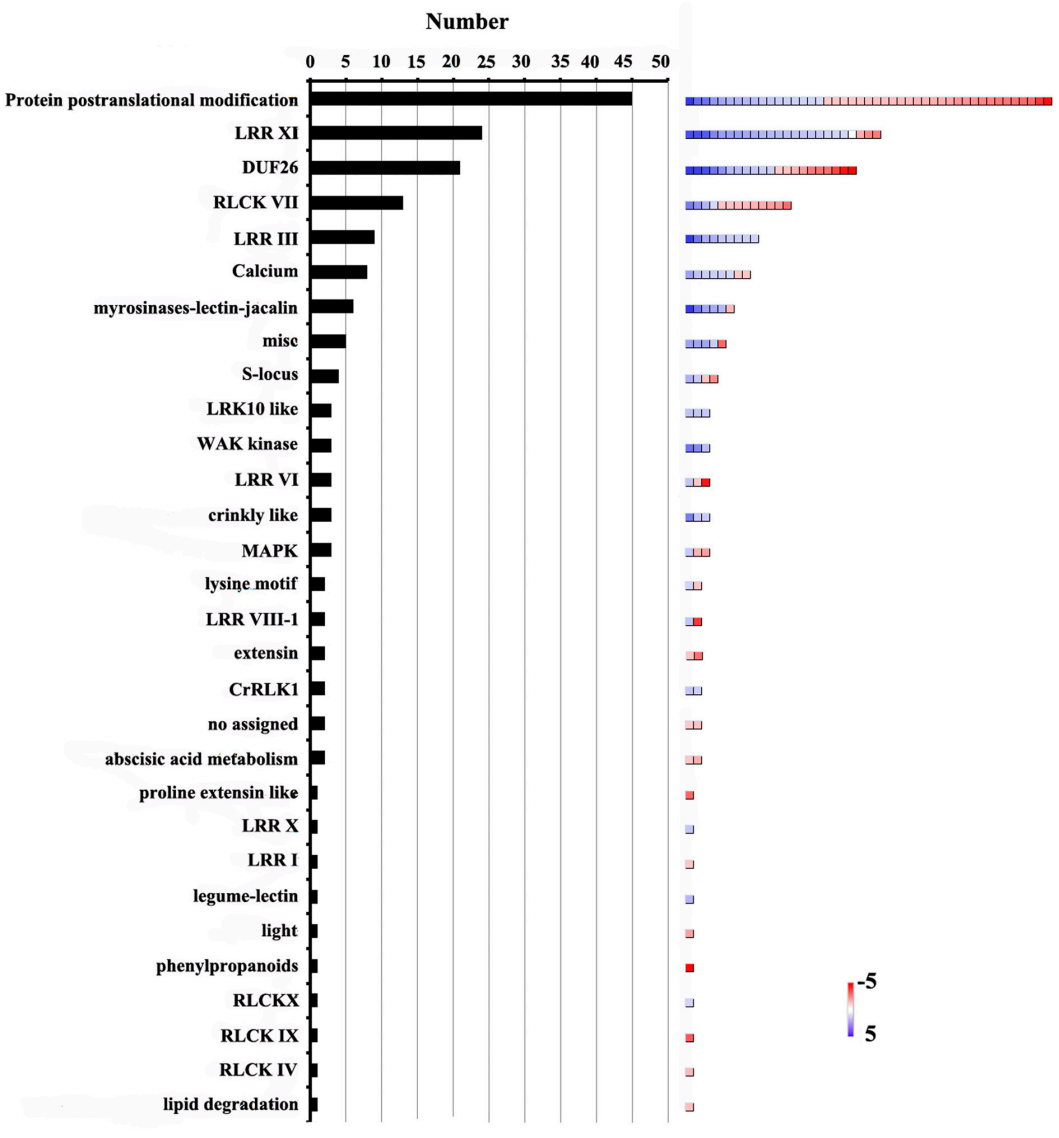
**Distribution of genes involved in protein amino acid phosphorylation among DEGs**. Number of genes in each category is shown on the left. Color scales (right) show relative transcript abundance of each gene (log_2_-transformed RPKM values) in the given categories.

The 172 genes involved in protein amino acid phosphorylation were further classified using MapMan software (Figure 6, Additional file 12). This analysis showed that the gene terms related to post-translational modification (26.1%), LRR-XI RLK (13.4%), DUF26 RLK (12.2%), RLCK VII (7.5%) and LRR-III RLK (5.2%), were overrepresented. Furthermore, 113 genes encoding receptor-like kinases (RLKs) were identified. These genes were further classified into 22 categories (Figure 6). The largest category consisted of genes encoding LRR-XI RLKs. These kinases are involved in various aspects of meristem development and nodulation (Brand et al., 2000; Krusell et al., 2002; Schnabel et al., 2005). Arabidopsis PEPR1 is a typical LRR receptor kinase with an extracellular LRR domain and an intracellular protein kinase domain. It belongs to the LRR-XI subfamily (Shiu et al., 2004). PEPR2 shares 76% amino acid similarity with PEPR1 (Yamaguchi et al., 2006). Both PERP1 and PERP2 could recognize damage associated with molecular patterns and active innate immunity pathways (Yamaguchi et al., 2006, 2010; Krol et al., 2010). Previous study proposed that conserved molecular components are involved in both pollen tube reception and fungal invasion in *Arabidopsis* (Kessler et al., 2010). In our data, the expression levels of 24 LRR-XI RLKs were significantly altered before and after pollination (Additional file 12). One of them, GRMZM2G428554 was homologous to PEPR1, implying that LRR-XI RKLs might be involved in the interaction between the embryo sac and the pollen tube in maize.

### Stress responses

**Figure 7:**
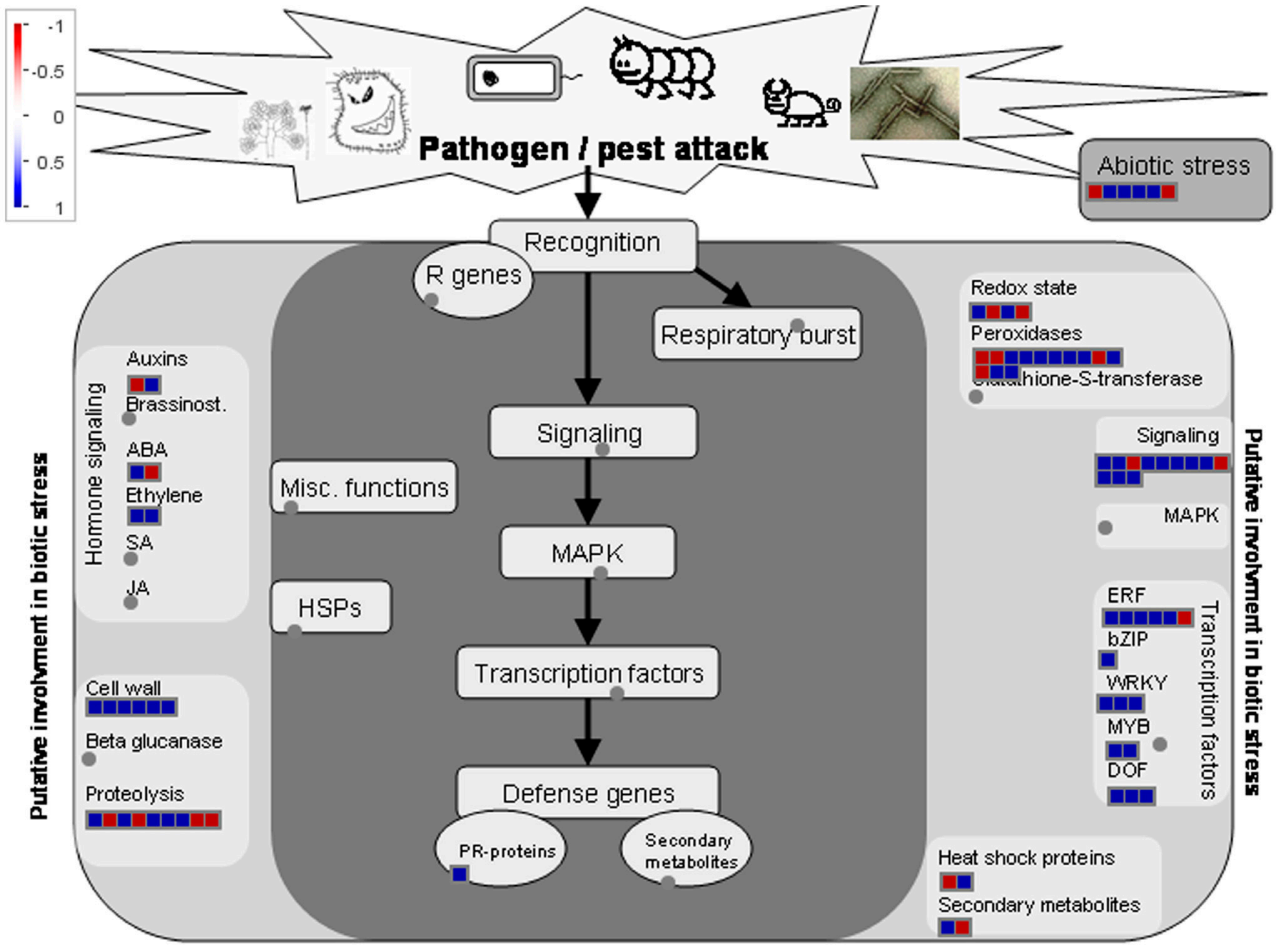
**Transcripts involved in stress responses**. Transcript expression levels represented by log_2_ transformed RPKM values of ESP relative to ES, The color of each box represents the level of up/down regulation of each transcript. Red represents down-regulation, blue represents up-regulation. The number of transcripts in each gene family is equal to the number of boxes in each gene family. The relative expression is represented by color scales as indicated.

It was suggested that there were similarities in genetic programs controlling pollination/fertilization and stress responses in rice (Lan et al., 2005). Here, totally we identified 214 genes related to stress responses in DEGs by GO analysis (Table 1, Additional 13). Of them, sixty-two genes were down-regulated and 152 genes were up-regulated, respectively. The 214 genes involved in stress responses were further classified using MapMan software (Figure 7). Most genes are involved in peroxidases, transcription factors, and signaling and cell wall. Stigmas have long been known to exhibit high levels of peroxidase activity when receptive to pollen (Dupuis and Dumas, 1990; Dafni and Motte Maues, 1998; McInnis et al., 2005). The Stigma-Specific Peroxidase (SSP) protein was identified and characterized in *Senecio squalidus* (McInnis et al., 2005). *Senecio* stigmas accumulate high levels of ROS in the epidermal cells (papillae) where SSP is localized (McInnis et al., 2006). In *Arabidopsis*, FER give rise to a high ROS environment at the entrance of the female gametophyte and it can mediate pollen tube rupture to release sperms for fertilization (Duan et al., 2014). In DEGs, *GRMZM2G100288* was homologous to *FER*, and there were 13 genes encoding the peroxidases, implying that ROS levels of embryo sac may have been changed after pollination (Additional file 13). Thus, the genetic network of stress response might also function in pollination in maize.

### Cysteine-rich peptides may regulate pollen tube guidance and embryo sac development

In plants, small secreted peptides (SSPs) play critical roles in defense, development, and many other physiological processes. Recent studies have revealed that various SSPs are involved in the pollen–pistil interaction. Interestingly, many of them are CRPs (Higashiyama, 2010; Chae and Lord, 2011). The maize CRP ZmES4 was shown to mediate pollen tube rupture by regulating the activity of the potassium channel protein KZM1 (Amien et al., 2010). Therefore, we speculated that CRPs may be involved in pollen tube guidance in maize. To test this hypothesis, we screened all the predicted proteins encoded by DEGs to identify proteins with a molecular weight of 20 kD or less, and identified 139 small proteins. Of them, 57 CRPs preferentially expressed in the ES (*vs*. ESP) were identified by local BLAST searches, using the cysteine arrangement model reported by Silverstein et al. (2007) as the search query. Using the same strategy, we identified 87 CRPs that were preferentially expressed in the ES (vs. Nu), and among them, 32 CRPs were preferentially expressed in the ES (vs. ESP) (Table 2). In total, 112 CRPs were identified among DEGs and EPGs.

**Table 2:** **Distribution of CRPs among DEGs and EPGs**.

**GENEID**	**Domain**	**Pattern Log_2_(ESP/ES)^a^**	**FDR**	**Pattern Log_2_(ES/Nu)^b^**	**FDR**
*GRMZM2G368861_T01*	Gamma-thionin			32.45	2.40E-08
*GRMZM2G101584_T01*	Gamma-thionin	−2.36	2.44E-74	10.84	0
*GRMZM2G047699_T01*	Gamma-thionin	1.19	1.35E-14	9.3	6.70E-92
*GRMZM2G047842_T01*	Gamma-thionin			9.77	3.00E-115
*GRMZM2G125520_T01*	Gamma-thionin	−1.66	5.61E-42	11.65	0
*GRMZM2G046532_T01*	Gamma-thionin			10.78	9.99E-176
*GRMZM2G012012_T01*	Gamma-thionin			36.79	4.28E-112
*GRMZM2G359064_T01*	Gamma-thionin			32.76	5.92E-10
*GRMZM2G128301_T01*	Gamma-thionin			36.08	5.86E-79
*GRMZM2G009359_T01*	Gamma-thionin			38.87	3.30E-248
*GRMZM2G357124_T01*	RALF			32.62	3.77E-09
*GRMZM2G383303_T01*	RALF	−1.77	2.70E-19	36.59	3.23E-102
*GRMZM2G056221_T01*	RALF			32.39	8.34E-08
*GRMZM2G424509_T01*	RALF			4.81	7.51E-60
*GRMZM2G153206_T01*	RALF	1.14	2.16E-20	2.44	2.13E-75
*GRMZM2G095039_T01*	RALF			1.13	1.95E-08
*GRMZM2G077259_T01*	RALF	1.1	8.17E-07	1.57	5.28E-09
*GRMZM2G301663_T01*	RALF	1.06	1.17E-10		
*GRMZM2G165083_T01*	Toxin_3	2.03	1.43E-15	33.54	5.15E-17
*GRMZM2G097084_T01*	Toxin_3			36.35	8.90E-91
*AC209356.4_FGT001*	Toxin_3	2.18	1.42E-12	32.81	1.74E-10
*AC199577.4_FGT004*	Toxin_3			38.29	1.03E-205
*GRMZM2G045082_T01*	Toxin_3			8.84	7.73E-180
*GRMZM2G171597_T01*	LTP_2	1.57	1.59E-13	7.42	1.77E-31
*GRMZM2G084325_T01*	LTP_2			8	7.25E-45
*GRMZM2G071575_T01*	LTP_2			3.97	1.44E-94
*GRMZM2G387360_T01*	LTP_2			4.25	1.70E-94
*GRMZM2G136364_T02*	LTP_2	4.52	4.16E-09		
*GRMZM2G126397_T01*	LTP_2	3.23	4.64E-42		
*GRMZM2G083725_T01*	LTP_2	2.79	8.66E-53		
*GRMZM2G078876_T01*	LTP_2	2.76	1.94E-103		
*GRMZM5G850455_T02*	LTP_2	2.57	3.55E-30		
*GRMZM2G104494_T01*	LTP_2	2.38	7.96E-08		
*GRMZM2G089400_T01*	LTP_2	1.48	9.88E-16		
*AC225127.3_FGT003*	Tryp_alpha_amyl			5.38	4.46E-08
*GRMZM2G404688_T01*	Tryp_alpha_amyl			32.06	1.90E-06
*GRMZM2G101958_T01*	Tryp_alpha_amyl			2.94	2.41E-83
*GRMZM2G406313_T01*	Tryp_alpha_amyl	32.18	2.49E-06		
*GRMZM2G010868_T01*	Tryp_alpha_amyl	6.29	5.38E-33		
*GRMZM5G898755_T01*	Tryp_alpha_amyl	−2.18	8.79E-09		
*GRMZM2G023847_T01*	Cu_bind_like	−3.95	5.24E-56	9.7	1.68E-111
*GRMZM2G146015_T01*	Cu_bind_like	1.04	7.93E-14	1.2	2.33E-15
*GRMZM2G004160_T01*	Cu_bind_like	−1.89	2.67E-05	2.54	1.50E-11
*GRMZM2G128531_T01*	Cu_bind_like			1.98	1.73E-23
*GRMZM2G027198_T01*	Cu_bind_like	1.22	2.37E-23		
*GRMZM2G317406_T01*	Pollen_Ole_e_I			32.67	2.04E-09
*GRMZM2G148064_T01*	Pollen_Ole_e_I			32.33	1.55E-07
*GRMZM2G173747_T01*	Pollen_Ole_e_I			2.08	2.14E-48
*GRMZM2G022347_T01*	Pollen_Ole_e_I	3.44	6.24E-07		
*GRMZM2G068202_T01*	GASA	1.39	2.80E-09	7.19	2.59E-27
*GRMZM2G172596_T01*	GASA	−2.64	2.21E-46	2.39	2.94E-49
*GRMZM2G117940_T01*	GASA	2.66	3.74E-43	7.29	3.50E-29
*GRMZM2G105364_T01*	GASA	2.3	2.35E-58		
*GRMZM2G077034_T01*	GASA	1.94	8.03E-26		
*GRMZM2G149869_T01*	SLR1-BP	−1.14	2.39E-16	38.56	3.92E-225
*GRMZM2G097719_T01*	SLR1-BP			34.77	1.72E-37
*GRMZM2G042125_T01*	Prolamin_like			10.88	1.39E-254
*GRMZM2G083190_T01*	Prolamin_like			9.19	3.80E-204
*GRMZM2G119812_T01*	Stig1	1.17	1.30E-06	7.21	8.75E-28
*GRMZM2G028266_T01*	Kazal_1			2.31	1.86E+01
*GRMZM2G156632_T01*	Bowman-Birk_leg			4.54	2.79E-27
*GRMZM2G041039_T01*	AmiS_UreI	1.38	6.43E-14	5.37	7.10E-50
*GRMZM2G146573_T01*	DUF1180			7.63	6.23E-88
*GRMZM2G129083_T01*	DUF1180			1.18	7.05E-14
*GRMZM2G079499_T01*	DUF3742			33.06	2.41E-12
*GRMZM2G050994_T01*	DUF2667			11.38	0
*GRMZM2G175165_T01*	DUF2667	−2	4.86E-55	13.08	0
*GRMZM2G038024_T01*	DUF1270	−1.27	2.01E-24	11.74	0
*GRMZM2G396540_T01*	DUF1218	1.85	7.63E-26		
*GRMZM5G872070_T01*	DUF1218	1.25	4.57E-10		
*GRMZM2G129189_T02*	Chitin_bind_1	−1.98	4.91E-07		
*GRMZM2G021621_T01*	DPBB_1	1.95	1.47E-25		
*GRMZM2G150172_T01*	NO			2.53	1.95E-66
*GRMZM2G014994_T01*	NO			2.33	2.00E-28
*GRMZM2G121256_T01*	NO			33.48	1.67E-16
*GRMZM2G136534_T03*	NO			2.82	6.69E-07
*AC212323.4_FGT005*	NO	1.16	2.12E-10	35.44	1.91E-55
*GRMZM2G044174_T01*	NO			2.32	4.07E-08
*GRMZM2G019644_T01*	NO			38.11	1.30E-193
*GRMZM2G112855_T01*	NO	−1.98	4.62E-41	38.46	4.03E-218
*GRMZM2G046252_T01*	NO	1.4	6.37E-21	36.5	1.24E-97
*GRMZM2G463167_T01*	NO	1.45	5.66E-20	36.05	1.04E-77
*GRMZM2G088169_T01*	NO	2.12	2.15E-36	8.24	7.95E-52
*GRMZM2G389937_T02*	NO	2.04	8.29E-09	32.51	1.29E-08
*GRMZM2G356256_T01*	NO	−2.42	5.68E-06	1.95	2.62E-07
*GRMZM2G039942_T01*	NO			10.19	5.58E-204
*GRMZM2G040098_T01*	NO			6.3	2.06E-29
*GRMZM2G118269_T02*	NO			34.39	1.21E-29
*GRMZM2G101553_T01*	NO			34.84	5.31E-39
*GRMZM2G118269_T01*	NO			34.45	8.51E-31
*GRMZM2G078799_T01*	NO			33.67	1.55E-18
*GRMZM2G040020_T01*	NO			34.3	8.75E-28
*GRMZM2G040020_T02*	NO			33.24	6.42E-14
*GRMZM2G006601_T01*	NO	−1.44	7.55E-33	12.3	0
*GRMZM5G878322_T01*	NO	1.2	2.92E-07	2.11	4.17E-12
*GRMZM2G145466_T01*	NO	−2.34	1.75E-68	39.9	0
*GRMZM2G483273_T01*	NO			11.97	0
*GRMZM2G489599_T01*	NO			11.72	0
*GRMZM2G074293_T01*	NO			11.99	0
*GRMZM2G483275_T01*	NO			10.5	0
*GRMZM2G489627_T01*	NO			9.48	1.05E-224
*GRMZM2G167151_T01*	NO			13.21	0
*GRMZM2G029926_T01*	NO			31.98	6.58E-06
*GRMZM2G079962_T01*	NO	−3.16	8.28E-140	12.35	0
*GRMZM2G055629_T01*	NO	−2.31	4.85E-11	7.27	5.17E-106
*GRMZM2G055629_T02*	NO			34.73	2.11E-36
*GRMZM2G180903_T02*	NO	−4.81	3.35E-07		
*GRMZM2G346499_T01*	NO	1.18	1.97E-12		
*GRMZM2G439337_T01*	NO	−1.68	3.06E-05		
*GRMZM2G166094_T01*	NO	1.12	9.51E-07		
*AC211652.4_FGT001*	NO	1.38	3.76E-07		
*GRMZM2G150688_T01*	NO	−2.78	1.64E-07		

a *Gene expression levels (log_2_-transformed RPKM values in ESP relative to ES)*.

b *Gene expression levels (log_2_-transformed RPKM values in ES relative to Nu)*.

CRPs can be grouped into classes according to their domains. The classes of the 112 CRPs were identified based on their conserved domains, using tools at the Conserved Domain database (http://pfam.sanger.ac.uk/search?tab=searchSequenceBlock) (Table 2). In previous studies, four maize CRPs containing the gamma-thionin domain (ZmES1, 2, 3, and 4) expressed in the embryo sac were shown to be involved in the embryo sac–pollen tube interaction (Cordts et al., 2001; Amien et al., 2010). In our dataset, GRMZM2G012012, GRMZM2G359046, GRMZM2G128301, and GRMZM2G009359 were identified as ZmES1, 2, 3, and 4, respectively, by BLAST sequence analysis. Pollen-specific rapid alkalinization factors (RALFs) function in pollen tube elongation (Covery et al., 2010). In our data set, three genes encoding maize RALFs (*GRMZM2G317406, GRMZM2G148064*, and *GRMZM173747*) were preferentially expressed in the ES. In lily, the stigma/stylar cysteine-rich adhesion (SCA) protein, a lipid-transfer protein containing a Tryp-alpha-amyl domain, was shown to be abundant in the stigma and the transmitting tract, and was responsible for pollen tube growth and guidance. Chemocyanin, belonging to the blue copper domain-containing protein family, was shown to induce pollen tube chemotropism, and its activity was enhanced by SCA (Kim et al., 2003, 2006; Lord, 2003; Park and Lord, 2003). In this study, there was one *SCA* homolog (*GRMZM2G101958*) among the DEGs. Among the EPGs, there were four genes (*GRMZM2G023847, GRMZM2G0146015, GRMZM2G0004160*, and *GRMZM2G1128531*) encoding products with a copper-binding protein-like (cu_bind_like) domain. One of them encoded a protein (GRMZM2G004160) showing 61.5% similarity to chemocyanin. Interestingly, the transcript level of *GRMZM2G0004160* differed markedly before and after the entry of the pollen tube. These results suggested that the interaction between SCA and chemocyanin might play an important role during pollination in maize.

The Cys-rich late anther tomato 52 protein (encoded by *LAT52*) was shown to be essential for pollen hydration and pollen tube growth. The LAT52 protein in tomato is an Ole e 1-like (Pollen Ole e I) domain-containing protein with a pollen-specific expression pattern (Muschietti et al., 1994; Jiang et al., 2005). Among the EPGs there were three genes (*GRMZM2G317406, GRMZM2G148064*, and *GRMZM173747*) encoding proteins with a Pollen Ole e I domain. One of them, *GRMZM2G148064*, was homologous to *LAT52*. A gene encoding a LAT52 homolog (*GRMZM022347*) was also present in the ESP/ES DEGs dataset. Two small secreted Brassica pollen coat proteins, SLR1-BP1 and SLR1-BP2, were shown to interact with SLR1 during pollen grain adhesion (Luu et al., 1999; Takayama et al., 2000). *GRMZM0149869* (corresponding to *SLR1-BP1*) and *GRMZM00977* were present in the EPGs dataset; the transcript level of *GRMZM0149869* was lower in the ESP than in the ES.

**Figure 8:**
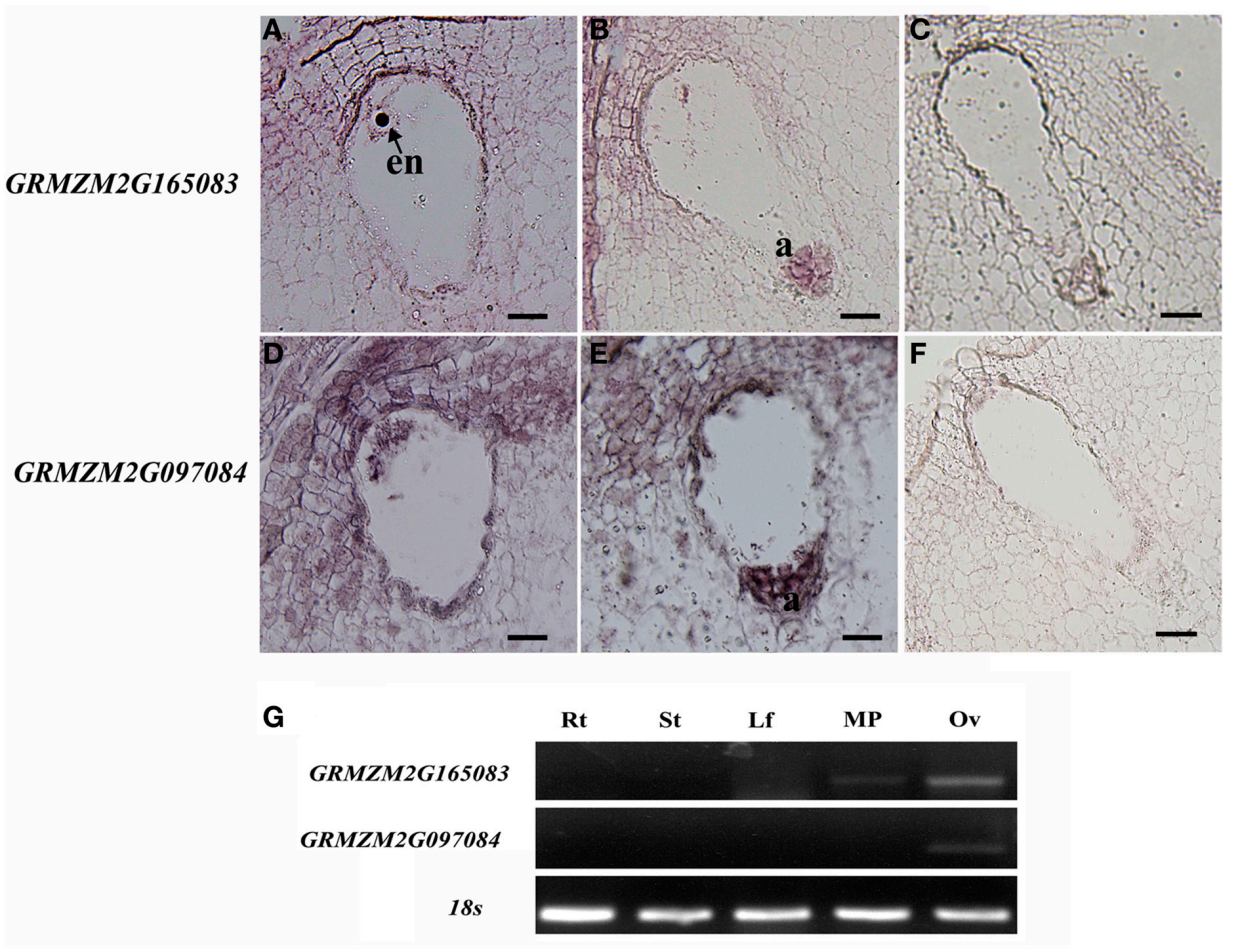
***In situ* hybridization patterns of *GRMZM2G165083* and *GRMZM2G097084*. (A,B)** *in situ* hybridization analysis of *GRMZM2G165083* expression with antisense probes; **(D,E)** *in situ* hybridization analysis of *GRMZM2G097084* expression with antisense probes; **(C,F)** sense probes. All micrographs show longitudinal sections of maize embryo sacs. **(G)** Semi-quantitative RT-PCR analysis of *GRMZM2G165083* and *GRMZM2G097084* transcript levels in maize tissues. en, egg nucleus; a, antipodal cells; Rt, root; St, stem; Lf, leaves; MP, mature pollen; Ov, ovary. Scale bars = 20 μm.

Animal toxins have been shown to modulate K^+^ channels, Na^+^ channels, or Ca^2+^-activated K^+^ channels either as pore blockers or as gating modifiers (Mouhat et al., 2004). Until now, there are a few studies on the CRPs with a Scorpion toxin-like domain (Toxin_3 domain) in plants. *AsG255* was expressed abundantly in nodules in *Astragalus sinicus*, and its protein was identified to contain a scorpion toxin-like domain at the C-terminus, which might function as the common signaling component involved in the plants' perception of soil microorganisms (Chou et al., 2006). Five genes encoding CRPs with toxin domains were present in the EPGs dataset (*GRMZM2G045082, GRMZM2G165083, GRMZM2G097084, AC199577.4*, and *AC209356.4*). All of these genes encoded proteins with a Toxin_3 domain. Two genes (*GRMZM2G165083* and *GRMZM2G097084*) were selected for *in situ* hybridization analysis in mature embryo sacs. The *GRMZM2G165083* mRNA accumulated in the egg cell, central cell, and antipodal cells (Figures 8A–C). *GRMZM2G097084* transcripts were detected in the embryo sac, and also in the integuments around the micropyle (Figures 8D–F). Semi-quantitative PCR analyses confirmed that the two genes were preferentially or specifically expressed in ovaries (Figure 8G). Further analysis showed the transcript levels of *GRMZM2G165083* and *AC209356.4* were higher in the ESP than in the ES in DEGs (Table 2). Together, these results suggested that toxin peptides might function in embryo sac development and/or in pollen tube guidance in maize.

### Validation of RNA-seq results

**Figure 9:**
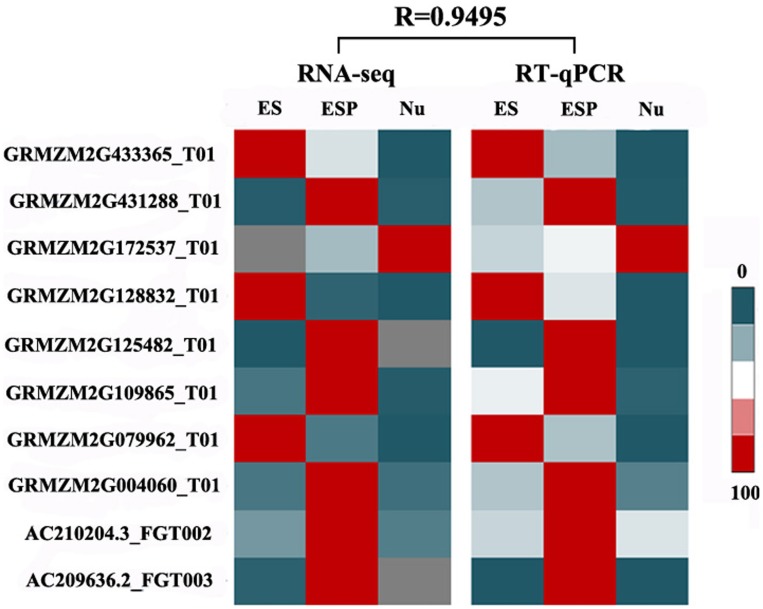
**Validation of RNA-seq results by RT-qPCR**. Transcript levels of 10 randomly selected genes in the four samples were detected by RT-qPCR. A heat map (center) was constructed using mean values of transcript levels detected in three biological replicates. Normalized RPKM values of RNA-seq results are shown (left). For each gene, maximum transcript level in a certain sample was set to 100, and transcript levels in other samples are shown relative to the maximum level. Relative transcript levels are shown by color scales (right). R, correlation coefficient value between RNA-seq data and RT-qPCR data.

To validate the RNA-seq analysis, ten genes were selected randomly for real-time qRT-PCR analyses. The details of these transcripts and their specific primers are shown in Additional file 2. The transcript profiles obtained by RT-qPCR were strongly correlated with those obtained in the RNA-seq analysis (*R* = 0.9495), confirming the reliability of the RNA-seq data (Figure 9).

## Conclusions and perspectives

In maize, the journey of the pollen tube toward the embryo sac can be divided into five phases. In phase I–III, the pollen grains adhere, hydrate and germinate on stigma cells. In phase IV, the pollen tubes grow between the sporophytic cell layers. In phase V, pollen tubes leave the sporophytic tissue and enter the ovary cavity (Heslop-Harrison et al., 1985; Johnson and Preuss, 2002; Lausser and Dresselhaus, 2010). During phases I–IV, the pollen tubes are controlled by signals from the sporophyte, however in phase V, they are controlled by signals from the gametophyte.

In the last decade or so, several studies have identified many molecules involved in pollen–pistil interactions and in the crosstalk between the male gametophyte and the female sporophyte. In our previous study, the transcript profiles of maize silks were analyzed at different developmental stages representing the most important events during pollination. Many genes related to microtubule-based movement, ubiquitin-mediated protein degradation, and transport were identified. These genes were involved in progamic pollen tube germination, adhesion, growth and guidance in phases I–IV (Xu et al., 2013). In phase V, species-specific signals or the barrier of the embryo sac itself are thought to control pollen tube growth and guidance in grasses. Thus, we conducted a preliminary analysis of transcripts involved in the embryo sac–pollen tube interaction. We identified 3467 DEGs between the ES and the ESP. Further analyses revealed that these DEGs may have roles in a number of biological pathways, including RNA binding, processing and transcription, miscellaneous enzyme family, signaling transduction, and protein targeting and degradation (Figure 3). The DEGs in the ES included genes encoding CRPs, such as ZMES4, ZMEA1, LURE1, and LURE2, and they function as key signaling factors in angiosperm reproduction (Márton et al., 2005; Dresselhaus and Márton, 2009; Okuda et al., 2009; Amien et al., 2010).

In conclusion, this RNA-seq dataset is an important resource for future study on maize reproduction. Further genetic and biochemical analysis will be critical to characterize their functions and to understand the mechanism of the maize embryo sac–pollen tube interaction.

### Conflict of interest statement

The authors declare that the research was conducted in the absence of any commercial or financial relationships that could be construed as a potential conflict of interest.
