# Supplementary figures and images for: Transcript profiles of maize embryo sacs and preliminary identification of genes involved in the embryo sac–pollen tube interaction
Source: Front Plant Sci. 2014 Dec 17;5:702. doi: 10.3389/fpls.2014.00702 (PMC4269116; doi:10.3389/fpls.2014.00702)

# Additional file 1

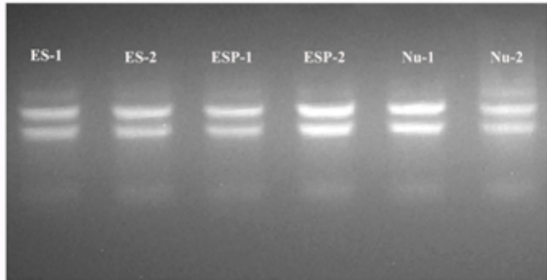

Supplement: Supplementary file 1 [file DataSheet1.PDF]

# Additional file 4

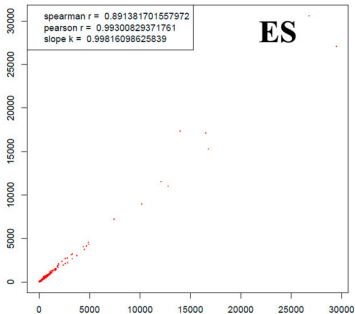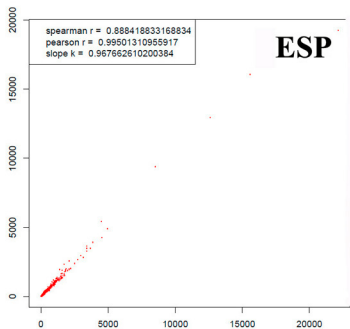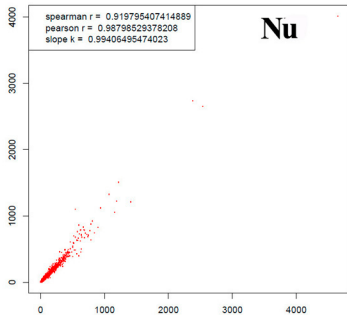

Supplement: Supplementary file 4 [file DataSheet4.PDF]
