# Supplementary material for: Transcript profiles of maize embryo sacs and preliminary identification of genes involved in the embryo sac–pollen tube interaction
Source: Front Plant Sci. 2014 Dec 17;5:702. doi: 10.3389/fpls.2014.00702 (PMC4269116; doi:10.3389/fpls.2014.00702)
Supplement: Supplementary file 2 [file DataSheet2.DOC]

| **Transcript ID** | **primer name** | **preimer sequence** |
| --- | --- | --- |
| GRMZM2G431288_T01 | GRMZM2G431288_T01-F | GTTGCTGTCCACTGTTCA |
|  | GRMZM2G431288_T01-R | ATTGATTACACCATTATTCCACTA |
| GRMZM2G079962_T01 | GRMZM2G079962_T01-F | ATTCTCACGAACAGTCAT |
|  | GRMZM2G079962_T01-R | TAACATTATACTATTGCCAGAG |
| GRMZM2G433365_T01 | GRMZM2G433365_T01-F | TTCAACGAGAACCAGAAG |
|  | GRMZM2G433365_T01-R | GGAAAACGATAGGATAAACC |
| AC209636.2_FGT003 | AC209636.2_FGT003-F | ACTCCTTCTACCTTAACA |
|  | AC209636.2_FGT003-R | GAGATGAAGCTCTTGATG |
| GRMZM2G125482_T01 | GRMZM2G125482_T01-F | GCGTGATTCTACAGCATA |
|  | GRMZM2G125482_T01-R | AACAACAGGAGCAATAAGT |
| GRMZM2G128832_T01 | GRMZM2G128832_T01-F | AACGATACCTCAGACTTG |
|  | GRMZM2G128832_T01-R | ATTATTTAGCCAGTGATTTACA |
| AC210204.3_FGT002 | AC210204.3_FGT002-F | AATCCCACCTAATCCACAT |
|  | AC210204.3_FGT002-R | TAACATCAACTATTCGCACAA |
| GRMZM2G109865_T01 | GRMZM2G109865_T01-F | GTACATCACCGGGAGCAG |
|  | GRMZM2G109865_T01-R | TACAGCCGATACATGCAC |
| GRMZM2G004060_T01 | GRMZM2G004060_T01-F | TCAGTACTCAGCAGCATACAT |
|  | GRMZM2G004060_T01-R | CTTGGGGTACGACGACAC |
| GRMZM2G172537_T01 | GRMZM2G172537_T01-F | AAGCCGAGATGTCATAGTAG |
|  | GRMZM2G172537_T01-R | GTAACGGCAATGGCAATG |

**Additional file 2**

A list of primers used in qRT-PCR.
