# Supplementary material for: Transcript profiles of maize embryo sacs and preliminary identification of genes involved in the embryo sac–pollen tube interaction
Source: Front Plant Sci. 2014 Dec 17;5:702. doi: 10.3389/fpls.2014.00702 (PMC4269116; doi:10.3389/fpls.2014.00702)
Supplement: Supplementary file 3 [file DataSheet3.DOC]

**Additional file 3**

**Distribution of reads sequenced from** the three studied samples（ES, PES and NU） **in maize reference genome and reference gene database.** (A) Summary of reads mapped to reference genome. (B) Summary of reads mapped to reference gene database.

(A)

| **Map to Genome** | **ES-1** | | **ES-2** | | **NU-1** | | **NU-2** | | **PES-1** | | **PES-2** | |
| --- | --- | --- | --- | --- | --- | --- | --- | --- | --- | --- | --- | --- |
|  | **reads number** | **ratio** | **reads number** | **ratio** | **reads number** | **ratio** | **reads number** | **ratio** | **reads number** | **ratio** | **reads number** | **ratio** |
| Total Reads | 9658379 | 100.00% | 9049923 | 100.00% | 9315667 | 100.00% | 9830584 | 100.00% | 6688964 | 100.00% | 6356811 | 100.00% |
| Total Base Pairs | 473260571 | 100.00% | 443446227 | 100.00% | 456467683 | 100.00% | 481698616 | 100.00% | 3.28E+08 | 100.00% | 3.11E+08 | 100.00% |
| Total Mapped Reads | 6888270 | 71.33% | 6489507 | 71.71% | 7345940 | 78.86% | 7782445 | 79.17% | 5372248 | 80.32% | 5098480 | 80.20% |
| perfect match | 4575841 | 47.38% | 4308481 | 47.61% | 5092929 | 54.67% | 5386099 | 54.79% | 3721192 | 55.63% | 3532667 | 55.57% |
| ≤3bp mismatch | 2312429 | 23.94% | 2181026 | 24.10% | 2253011 | 24.19% | 2396346 | 24.38% | 1651056 | 24.68% | 1565813 | 24.63% |
| unique match | 5923930 | 61.33% | 5560581 | 61.44% | 6331807 | 67.97% | 6713113 | 68.29% | 4684724 | 70.04% | 4441529 | 69.87% |
| multi-position match | 964340 | 9.98% | 928926 | 10.26% | 1014133 | 10.98% | 1069332 | 10.88% | 687524 | 10.28% | 656951 | 10.33% |
| Total Unmapped Reads | 2770109 | 28.68% | 2560416 | 28.29% | 1969727 | 21.14% | 2048139 | 20.83% | 1316716 | 19.68% | 1258331 | 19.80% |

(B)

| **Map to Gene** | **ES-1** | | **ES-2** | | **NU-1** | | **NU-2** | | **PES-1** | | **PES-2** | |
| --- | --- | --- | --- | --- | --- | --- | --- | --- | --- | --- | --- | --- |
|  | **reads number** | **ratio** | **reads number** | **ratio** | **reads number** | **ratio** | **reads number** | **ratio** | **reads number** | **ratio** | **reads number** | **ratio** |
| Total Reads | 9658379 | 100.00% | 9049923 | 100.00% | 9315667 | 100.00% | 9830584 | 100.00% | 6688964 | 100.00% | 6356811 | 100.00% |
| Total Base Pairs | 4.73E+08 | 100.00% | 4.43E+08 | 100.00% | 4.56E+08 | 100.00% | 4.82E+08 | 100.00% | 3.28E+08 | 100.00% | 3.11E+08 | 100.00% |
| Total Mapped Reads | 6806636 | 70.47% | 6289730 | 69.50% | 7121364 | 76.45% | 7528825 | 76.59% | 5480964 | 81.94% | 5212981 | 82.01% |
| perfect match | 4734301 | 49.02% | 4370501 | 48.29% | 5159052 | 55.38% | 5443973 | 55.38% | 3937644 | 58.87% | 3746188 | 58.93% |
| ≤2bp mismatch | 2072335 | 21.46% | 1919229 | 21.21% | 1962312 | 21.06% | 2084852 | 21.21% | 1543320 | 23.07% | 1466793 | 23.07% |
| unique match | 3259492 | 33.75% | 3021868 | 33.39% | 3005738 | 32.27% | 3199198 | 32.54% | 2680125 | 40.07% | 2540647 | 39.97% |
| multi-position match | 3547144 | 36.73% | 3267862 | 36.11% | 4115626 | 44.18% | 4329627 | 44.04% | 2800839 | 41.87% | 2672334 | 42.04% |
| Total Unmapped Reads | 2851743 | 29.53% | 2760193 | 30.50% | 2194303 | 23.55% | 2301759 | 23.41% | 1208000 | 18.06% | 1143830 | 17.99% |
